# The Structural–Functional Crosstalk of the Calsequestrin System: Insights and Pathological Implications

**DOI:** 10.3390/biom13121693

**Published:** 2023-11-23

**Authors:** Chiara Marabelli, Demetrio J. Santiago, Silvia G. Priori

**Affiliations:** 1 Department of Molecular Medicine, University of Pavia, 27100 Pavia, Italy; silvia.priori@icsmaugeri.it; 2 Laboratory of Molecular Cardiology, IRCCS ICS Maugeri, 27100 Pavia, Italy; 3 Centro Nacional de Investigaciones Cardiovasculares Carlos III (CNIC), 28029 Madrid, Spain; demetriojulian.santiago@cnic.es

**Keywords:** calsequestrin, calcium, intracellular ion channels, ryanodine receptor, ion channel regulators, calcium-binding proteins, polymerization disorders, arrhythmias, tubular aggregate myopathy, malignant hyperthermia

## Abstract

Calsequestrin (CASQ) is a key intra-sarcoplasmic reticulum Ca^2+^-handling protein that plays a pivotal role in the contraction of cardiac and skeletal muscles. Its Ca^2+^-dependent polymerization dynamics shape the translation of electric excitation signals to the Ca^2+^-induced contraction of the actin-myosin architecture. Mutations in CASQ are linked to life-threatening pathological conditions, including tubular aggregate myopathy, malignant hyperthermia, and Catecholaminergic Polymorphic Ventricular Tachycardia (CPVT). The variability in the penetrance of these phenotypes and the lack of a clear understanding of the disease mechanisms associated with CASQ mutations pose a major challenge to the development of effective therapeutic strategies. In vitro studies have mainly focused on the polymerization and Ca^2+^-buffering properties of CASQ but have provided little insight into the complex interplay of structural and functional changes that underlie disease. In this review, the biochemical and structural natures of CASQ are explored in-depth, while emphasizing their direct and indirect consequences for muscle Ca^2+^ physiology. We propose a novel functional classification of CASQ pathological missense mutations based on the structural stability of the monomer, dimer, or linear polymer conformation. We also highlight emerging similarities between polymeric CASQ and polyelectrolyte systems, emphasizing the potential for the use of this paradigm to guide further research.

## 1. An Introduction to CASQ

### 1.1. The Right Buffer at the Right Place

**Figure 1:**
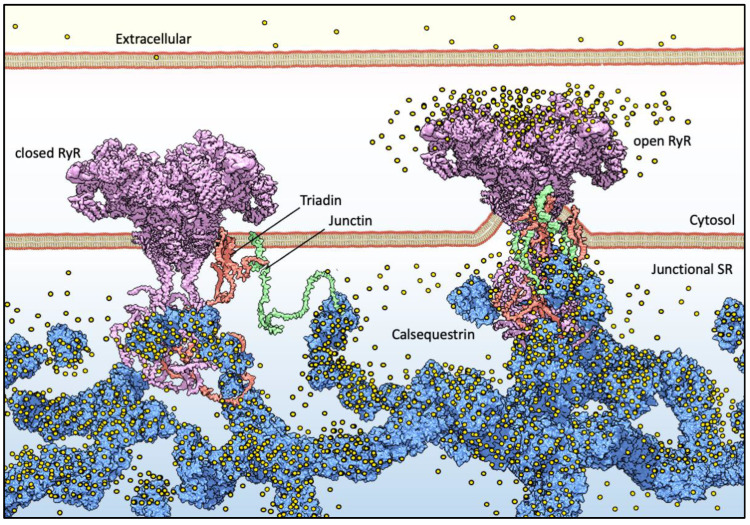
Basic components of Ca^2+^ release machinery in striated muscles. Excitation–contraction coupling (ECC) is the process by which an action potential at the sarcolemma leads to a massive release of intracellular Ca^2+^ which, in turn, activates cell-wide contraction. ECC occurs at subcellular structures that are periodically distributed inside muscle cells, called “junctions” (when referred to in structural terms) or Ca^2+^ Release Units (CRUs, when referred to in functional terms). Junctions are so termed because they are composed of specialized regions of the sarcolemma and of the “junctional” sarcoplasmic reticulum (jSR), both coalescing within nanometer distances. ECC occurs via different mechanisms in skeletal and cardiac muscles, so in the figure, the sarcolemmal portion of the junctions is not decorated with tissue-specific components. At the jSR membrane, CRUs are decorated by ordered arrays of ryanodine receptors (RyR, pink), which act as the SR Ca^2+^ release channels. In the lumen of the jSR, CASQ (blue) buffers Ca^2+^ ions (yellow spheres), whereas Junctin (green) and Triadin (red) are transmembrane proteins anchoring CASQ to the RyR and acting as signaling mediators between CASQ and RyR. The actual stoichiometry for the Jnt:Trd:RyR complex is unknown.

For muscle sarcomere contraction to occur, large quantities of Ca^2+^ must flow out of the sarcoplasmic reticulum (SR) lumen into the cytosol, driven by a very steep electrochemical gradient (SR/cytosol Nernst potential for Ca^2+^ is approx. 125 mV in a non-contracting cell). The maintenance of this on-demand Ca^2+^ supply partially relies on the highly acidic calsequestrin (CASQ) protein, and its interaction with each member of the Calcium Release Unit (Figure 1): Junctin (JNT), Triadin (TRDN), and RyR [1,2,3]. CASQ is relatively small (45 kDa), and yet is the most prominently expressed protein within the lumen of the “junctional” sarcoplasmic reticulum (jSR) (up to 100 mg/mL) [4,5]. Abundant quantities of Ca^2+^ cooperatively bind to CASQ with low affinity, allowing for a rapid back-and-forth (with each contraction–relaxation cycle) exchange of massive quantities of Ca^2+^ between CASQ and the matrix of the jSR. In mammals, two CASQ genes are differentially expressed in skeletal fast-twitch fibers (CASQ1) and cardiac muscle (CASQ2), whereas both isoforms are equally represented in slow-twitch fibers [6,7]. Measurements in CASQ2-KO mice indicate that CASQ2 stores about 50% of the cardiac SR Ca^2+^ content [8] while maintaining a free calcium concentration of approximately 1 mM [9]. Similar studies in CASQ1-KO fast-twitch skeletal muscles indicate that the Ca^2+^ stored in CSQ1 contributes 75% of the released calcium [10], while still maintaining a free Ca^2+^ concentration of approximately 1 mM in the resting fiber.

### 1.2. CASQ Macro-Architecture(s)

**Figure 2:**
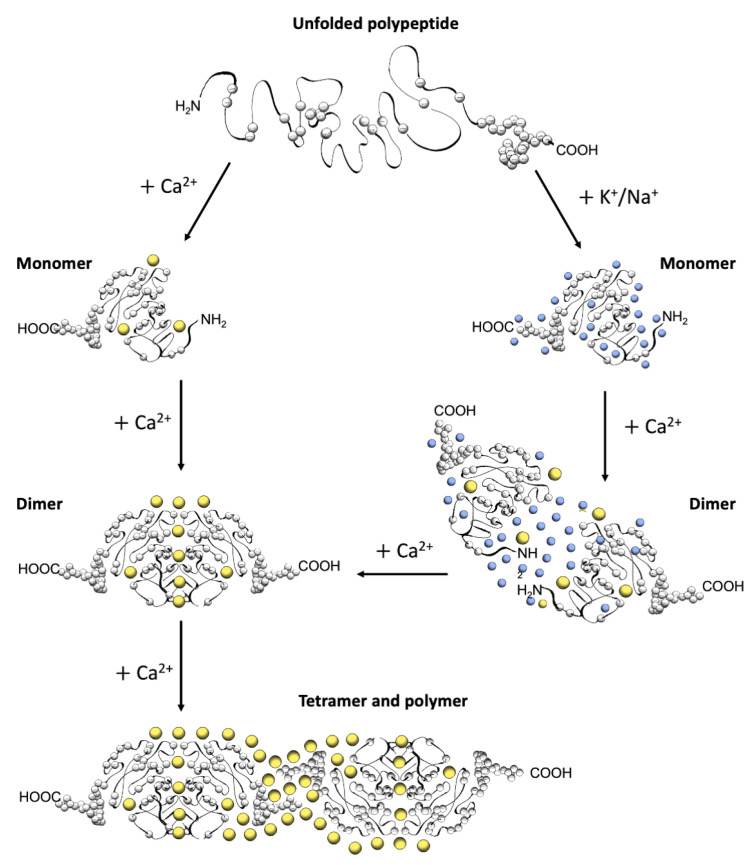
Schematic diagram of CASQ structural transitions guided by increasing amounts of calcium and/or monovalent cations. Negative charges along the polypeptide backbone are represented as white spheres. At close-to-neutral pH and low ionic strength, CASQ retains an unfolded conformation. Either monovalent ions such as Na^+^ or K^+^ (blue spheres) or divalent ions such as Ca^2+^ (yellow spheres) mask the negative charges of the abundant exposed glutamate and aspartate residues (103 and 102 acidic residues over 362 (CASQ1) and 380 (CASQ2) amino-acid-long polypeptides, respectively). In both cases, the ionic strength increase triggers formation of the same tertiary structure. To this end, the concentrations of Ca^2+^ required are about three orders of magnitude lower (about 0.1 mM) than that of other monovalent cations (100 mM). Further Ca^2+^ addition triggers dimerization through N-terminal tail swapping. A higher Ca^2+^ concentration is required for tight dimerization to occur when competing Na^+^ and K^+^ ions are present. The further increase in Ca^2+^ ions promotes “back-to-back” tetramerization, which concomitantly leads to an enormous increase in the calcium-binding capacity of each CASQ monomer. The protein’s intrinsically disordered C-termini, involved in stabilizing the Ca^2+^-dependent tetramer, are represented in a fixed conformation for clarity of representation.

The first electron micrographs of the jSR revealed an electron-dense filling able to assume the most diverse conformations from wire-shaped structures immediately beneath and parallel to the junctional membrane to intricately branched filaments and spherical bodies or puncta [11,12,13,14,15,16,17]. Imaging in vivo and cross-linking experiments support the notion that this material is Ca^2+^-complexed CASQ [18,19,20]. In addition, these structures disassemble and disperse under luminal Ca^2+^ depletion conditions [15,21], which correlates with the fact that the recombinant protein can organize in vitro into multiple architectures of varying compactness, hierarchically ordered in response to rising Ca^2+^ concentrations from micromolar levels up to 20 mM [3,16,17,22] (Figure 2). As higher order polymeric structures are formed, the capacity and cooperativity of Ca^2+^-binding events show a parallel, stepwise increase, which has consequences for the modulation of intra-SR Ca^2+^ storage and release. But how are CASQ multimers organized?

## 2. CASQ Multimeric Organization

### 2.1. CASQ Secondary and Tertiary Folding Is Sustained by Cations

**Figure 3:**
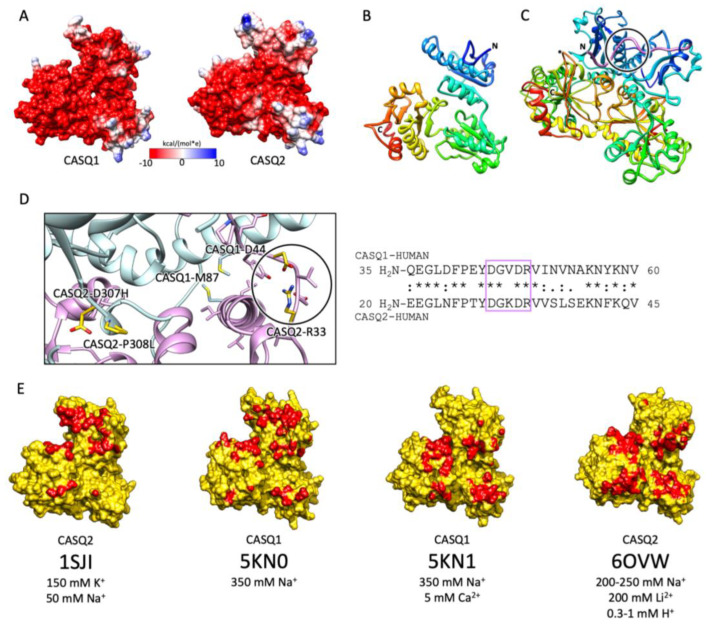
CASQ monomeric structure and salt-sensitive dimerization. (**A**) Representation of skeletal (left, PDB ID: 5KN0) and cardiac (right, PDB ID: 1SJI) CASQ surfaces. Colors mirror the coulombic potential values according to the color legend shown. The disordered C-terminus is not present in the experimental structures. (**B**) Ribbon representation of CASQ monomer. The PDB model is 1SJI for cardiac CASQ2. The polypeptide is conventionally rainbow-colored starting from its N-terminus (blue) to its C-terminus (red). (**C**) The corresponding cardiac CASQ dimer is shown (PDB ID: 1SJI). One of the two swapped N-terminal domains within the dimeric state is colored violet for ease of identification. The N-terminal portion within the circle corresponds to the dimerization switch. (**D**) Left: Zoomed view of the dimerization N-terminal switch (within the circle). The most relevant pathological missense mutation residues falling close or within the dimerization interface are evidenced in gold and represented as sticks. Right: The sequences of the N-termini for both skeletal and cardiac CASQ are compared. Identical or similar residues are evidenced by an asterisk or a colon, respectively. The dimerization switch is highlighted in the violet box. (**E**) Surface representation of single monomers, where the dimerization interface is, is evidenced in red. For each structure, the concentrations of the cations of crystallization conditions are indicated below the relative PDB ID.

A high abundance of carboxylates, carried by aspartic and glutamic acids, characterizes all CASQ isoforms, with the human skeletal and cardiac proteins featuring an isoelectric point of 4.0 and 4.2, respectively (Figure 2). Due to repulsion between negative charges, the polypeptide retains an extended, random coil conformation at low ionic strength (i.e., lower than that provided by 100 mM KCl) [23]. Multiple, monovalent, or divalent cations can likewise guide the folding of three negatively charged, nearly identical, thioredoxin domains (Figure 3A,B) [24,25,26,27,28], where numerous hydrophobic interactions hold the interior of the domains [29]. The minimal ionic concentration sustaining secondary and tertiary protein folding varies with the coordination number and ionic radius of the cation [23,24,25,27,28,29,30], with Ca^2+^ featuring the lowest effective concentrations, plus distinctive binding cooperativity even at modest ionic strength [23,25]. Low concentrations of monovalent cations (i.e., 85–150 mM KCl) have a cooperative effect on Ca^2+^-binding events at the early stages of protein folding [23,29,31]. It appears, however, that similar or higher abundances of monovalent ions inhibit compaction of the critical, dimerization-competent monomer, which is ultimately stabilized by a minimal amount of Ca^2+^ [32].

### 2.2. The Ca^2+^-Specific Dimerization Switch Is Salt-Sensitive

The first and obligatory step of CASQ supra-molecular assembly is the Ca^2+^-driven “front-to-front” dimerization through N-terminal domain swapping (Figure 3C,D) [29,32,33,34]. Upon Ca^2+^ binding, a few alternatively charged residues of the N-terminus flip and establish a hydrogen bonding network. In turn, this secures the extended conformation of the N-terminal tail over the surface of the opposite monomer (Figure 3C,D). Multiple intercalated Ca^2+^ ions bridge in between the abundant carboxylate groups exposed from each monomer’s surface, as revealed by equilibrium dialysis [4,32]. The comparison between different crystal structures of the CASQ dimers reveals that the inter-monomer space could also be filled by other cations than Ca^2+^. Nonetheless, only divalent ions engage the two monomers in a tight architecture, allowing more hydrophobic residues to line the inter-monomer interface (Figure 3E) [31,32,35,36].

### 2.3. Poorly Understood Ca^2+^-Dependent Mechanisms Drive CASQ Polymerization

**Figure 4:**
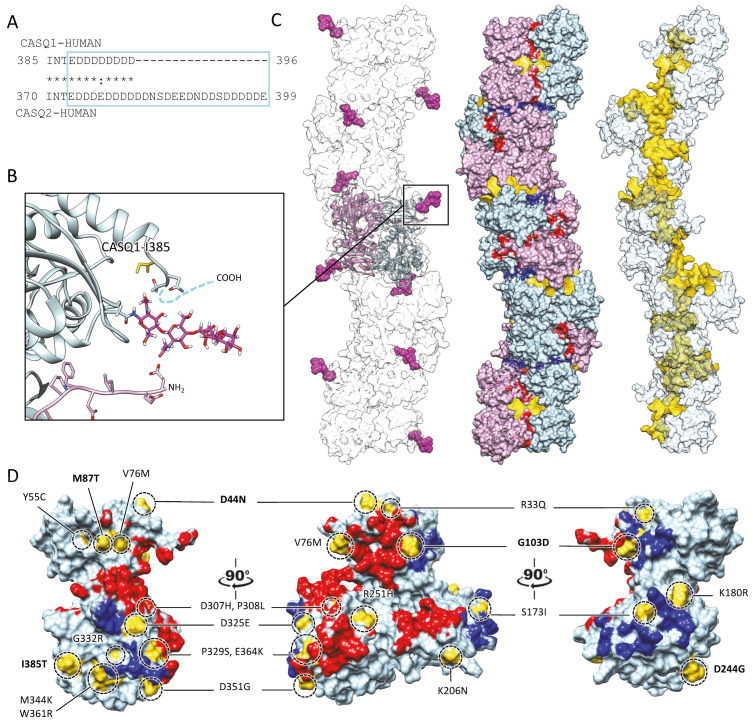
Multiple sites globally design the quaternary assembly of CASQ. (**A**) The C-terminal Intrinsically Disordered Region is highlighted in the blue box for skeletal and cardiac CASQ isoforms. (**B**) Inset from the glycosylated skeletal CASQ dimer structure (PDB ID: 3TRQ), showing the relative positions and orientations of the glycosyl moieties on Asn-316, with respect to the C-terminus of the glycosyl-carrying monomer and the N-terminus of the dimeric partner. The presence of a two-mannosyl chain is sufficient to fully stabilize the N-terminus, whereas the C-terminus retains a disordered conformation. The site of the known pathological missense mutation I385T in CASQ1 is close to the glycosylation site. (**C**) CASQ polymeric structure is represented for a portion corresponding to five dimers (PDB ID 6OWW). Left: The structure of native glycosylated skeletal CASQ1 (PDB ID: 3TRQ) is fitted within the 6OWW polymeric structure to visualize the position of the glycosyl moieties (magenta) with respect to the linear polymer surface (transparent). Centre: For each dimer, one monomer is colored light blue, and the second monomer is colored pink. A 90° rotation from one dimer to the adjacent one is evident along the polymer axis. The inter-monomer surfaces are colored red, whereas the inter-dimer interfaces are colored blue. The empty tunnel running within the structure is colored yellow. Entry/exit spaces in continuity with the internal tunnel are visible. Right: The internal tunnel is shown in yellow, and only one monomer for each dimer (corresponding to light blue monomers in the central polymer structure) is represented as a semi-transparent surface. (**D**) Surface missense pathological mutations are evidenced in gold on the surface of a CASQ2 monomer (PDB ID: 6OWW). The inter-monomer surfaces are colored red, whereas the inter-dimer interfaces are colored blue. CASQ1 mutations (D44N, M87Q, G103D, D244G, and I385T) are in bold for ease of identification among the numerous CASQ2 missense mutations.

The bottleneck of CASQ polymerization is proper tetramer assembly [4,32]. The ordered “back-to-back” tetramerization necessitates the intrinsically disordered C-terminal tail [37,38]. Truncation mutants lacking the C-terminus indeed behave as constitutive dimers in the solution and fail to multimerize upon Ca^2+^ addition [4]. It has been hypothesized that the strong negatively charged tail repels the formation of improperly positioned dimers, yet the exact mechanism driving physiological polymerization has not been elucidated. Interestingly, the main difference between skeletal and cardiac isoforms lies precisely in the C-terminal segment (Figure 4A). The longer and more negatively charged C-terminus of CASQ2 drives multimerization at higher Ca^2+^ concentrations (~2 mM) than CASQ1 (~0.7 mM) [37,38], and the swapping of this segment between isoforms causes the reciprocal exchange of their Ca^2+^-dependent polymerization kinetics [37]. As the coulombic properties of the C-terminal tail drive the specific conformational responsiveness to Ca^2+^, the other negative charges of the polypeptide shape the surface electric potential of the growing CASQ polymer, with CASQ1 polymers providing a more charged surface than CASQ2, onto which more Ca^2+^ ions can be adsorbed [4]. Since the dimer-to-tetramer transition, CASQ Ca^2+^ binding capacity and cooperativity increase; newly bound Ca^2+^ ions somehow stabilize further Ca^2+^-coordinating sites. The outcome is a multimeric species endowed with an enormous Ca^2+^-binding capacity; up to 60 Ca^2+^ ions are bound per molecule for human cardiac CASQ2, and up to 80 for the skeletal CASQ1 [4,29,32].

## 3. CASQ Polymer Dynamics Influence Ca^2+^ Release from jSR

### 3.1. CASQ Is a Dynamic System

While there is no question that CASQ forms polymers in vivo, the extent to which depolymerization–polymerization cycles occur during a single Excitation–Contraction Coupling (ECC) cycle is a matter of ongoing research. The shifts in the equilibrium among distinct CASQ quaternary states have huge functional implications both directly on the Ca^2+^ storage capacity and Ca^2+^ release kinetics, either indirectly through affecting the protein’s localization and interactome. In skeletal muscle, limited changes in intra-SR Ca^2+^ weaken CASQ1 architecture and increase its mobility within the longitudinal SR [16]. In cardiac muscle, beat-to-beat variations in CASQ2 multimerization status have not been experimentally verified, to our knowledge. Considering the effect of post-translational modifications (which will be discussed in Section 4), it seems likely that CASQ2 (with a measured in vitro Ca^2+^ affinity for the recombinant form of about 2 mM [4,29,37]) at least partially polymerizes in vivo at the physiological 1 mM intra-SR Ca^2+^. Further in silico studies exploring this possibility rendered that such quaternary state transitions would be important in shaping Ca^2+^ release termination and refractoriness [39]. Still, dissecting the direct effect of CASQ on RyR activity versus the indirect effects on intra-SR Ca^2+^ buffering (which in turn also affects RyR activity) remains a technically challenging question.

### 3.2. CASQ Ca^2+^-Driven States Physically Tune the Ca^2+^ Release Channel

Both in skeletal [16] and cardiac [40,41] muscles, CASQ interplays with the JNT-TRD-RyR Ca^2+^ Release Unit (CRU) (Figure 1) [42]. Two SR transmembrane proteins, TRDN and JNT, are essential, non-interchangeable mediators of the CASQ-dependent modulations of RyR activity [40,43,44,45,46]. JNT and TRDN both feature a single-transmembrane helical domain and a long, flexible, luminal domain exposing multiple KEKE (Lys-Glu-Lys-Glu) motifs, believed to be CASQ-anchoring points [3,47]. CASQ recruitment by TRDN and JNT is in bidirectional equilibrium with its Ca^2+^-dependent polymerization. Both JNT and TRD stabilize CASQ polymeric structures in vivo [1,48,49], whereas in vitro, varying stoichiometries of JNT shape CASQ2 polymerization dynamics [3,50]. On the other hand, the CASQ Ca^2+^-driven conformational state affects its affinity for JNT and TRD; C-terminally phosphorylated CASQ1, which polymerizes at lower Ca^2+^ than its unphosphorylated form, is more readily recruited by Junctin [3,51,52]. Similarly, the strength of CASQ–RyR interaction is also Ca^2+^-sensitive; it remains intact for free Ca^2+^ levels within the physiological SR range (0–1 mM) but diminishes at 5 mM Ca^2+^ [53]. Regarding the identification of the CASQ-binding region of RyR, it has been revealed that, when CASQ2 is co-expressed in HEK293 cells alongside RyR (HEK293 cells are heterologous expression systems which do not contain detectable levels of neither endogenous CASQ nor endogenous RyR), CASQ2 can directly bind the first luminal loop (residues 4521–4573) of the cardiac RyR2 [53]; yet, different in vitro tests could not identify a critical region for such binding [54]. Interestingly, the C-terminal domain of CASQ serves as a crucial mediator (although not the sole one) for its interaction with any component of the CRU [45,55]. The particular functional impact of CASQ on RyR activity is determined by the specific partner involved, where TRDN and JNT, despite their similarities, mediate distinct effects on the opening and closure of the RyR channel [49,56,57,58].

### 3.3. CASQ Polymerization Regulates ECC Indirectly by Modifying Free Intra-SR Ca^2+^

Bilayer [40,59] and in vitro [8,60] data suggest that, in the absence of CASQ, RyR activity is still sensitive to varying intra-SR Ca^2+^ levels. Cardiac RyRs are endowed with activating intra-SR Ca^2+^-binding sites [59], whereas skeletal RyRs feature a Ca^2+^-dependent inactivation site on their cytosolic side [61]. Whatever the mechanism of release modulation, what is clear is that CASQ-independent mechanisms exist intrinsic to the RyRs which are able to activate and terminate SR Ca^2+^ release. Moreover, intra-SR Ca^2+^ buffering via distinct CASQ quaternary states, together with proper CASQ retention at the jSR, are essential for indirectly shaping the physiological kinetics of jSR Ca^2+^ release and refractoriness (i.e., the ability for RyRs to activate a second time once they have been already active). Accordingly, CASQ2-related hereditary cardiac arrhythmias commonly feature an impaired release refractoriness [8], whereas mutations or deletions of skeletal CASQ1 have been linked to contraction defects, fatigue [16], and degenerative skeletal muscle diseases [15].

Finally, it must also be pointed out that CASQ is neither the only intra-SR Ca^2+^ buffer nor the only intra-SR Ca^2+^ release modulator. The histidine-rich calcium-binding protein (HRC) is a structurally distinct, high capacity, low Ca^2+^ affinity buffer (HRC KD = 1.9 mM [62]). Since no Ca^2+^-binding motifs are known within HRC, it is presumed that its acidic repeats constitute its Ca^2+^ binding sites [63] (HRC contains more acidic sites than CASQ). Functional studies in CASQ2/HRC double-KO mice have revealed that the absence of both proteins improves the cardiac arrhythmic phenotype vs. CASQ2-KO mice [64]. Since HRC, like CASQ, has the capability of binding TRDN in a Ca^2+^-dependent manner, a model has been proposed in which the CASQ2-dependent and HRC-dependent regulation mechanisms of SR Ca^2+^ release are complementary; TRDN would bind CASQ2 at low intra-SR Ca^2+^ levels (inhibiting the channel), and HRC would bind TRDN at high SR-free Ca^2+^ (priming the channel for further aperture) [64]. Besides these direct and indirect effects on SR Ca^2+^ release, HRC has been shown to modulate SR Ca^2+^ uptake in a SR Ca^2+^-dependent manner by directly binding to SERCA [65]. The phosphorylation of HRC S96 by Fam20C regulates the interactions of HRC with both TRDN and SERCA2a [66]. Consequently, the HRC mutation S96A (S81A in mice) has been linked to arrhythmias and sudden cardiac death [67].

## 4. Post-Translational Modifications Modulate the Responsivity of the CASQ System

### 4.1. CASQ Dynamics Are Post-Translationally Tuned

The Ca^2+^-binding and multimerization properties of CASQ are tightly and intimately related to its trafficking and retainment at the jSR. Both pathological missense mutations and N-terminal tags alter the protein’s physiological localization [68,69,70,71]. It stands to reason that post-translational modifications of CASQ, by shaping the kinetics of Ca^2+^-dependent quaternary assembly, also modulate CASQ trafficking and retention within the jSR. Multiple covalent modifications at various sites have been detected using mass-spectrometry including glycosylation, phosphorylation, and even the proteolysis of its N-terminal segment [34,72,73]. Above all, the favored modification hotspot is the C-terminus. This further highlights the critical and intertwined role of this disordered segment in modulating the protein’s Ca^2+^-related properties.

### 4.2. Phosphorylation

Phosphorylation at either Thr-353 by Casein Kinase 2 [51,52] or at Ser-248 and Ser-369 by Fam20C [21] increases the responsiveness to Ca^2+^ of recombinant skeletal CASQ1 [72]. Likewise, for cardiac CASQ2, the phosphorylation of Ser-385 by Fam20C increases its overall Ca^2+^-binding capacity [74], whereas the phosphorylation of both Ser-385 and Ser-393 by Casein Kinase 2 lowers the Ca^2+^ threshold for polymerization in vitro [72]. The augmented sensitivity of phosphorylated CASQ1 and CASQ2 to Ca^2+^ could be explained, in principle, by the higher chemical affinity of Ca^2+^ towards phosphate rather than carboxylate groups. This mechanism may not be unique to CASQ, as the phosphorylation of the highly flexible protein Casein increases its Ca^2+^-binding capacity, its solubility, and its ability to form Ca^2+^-containing colloidal suspensions [75,76]. It appears that the C-terminus of skeletal CASQ1 is mainly unphosphorylated in vivo, whereas variable amounts of C-terminally non-phosphorylated, singly phosphorylated, and doubly phosphorylated CASQ2 have been confirmed to co-exist in cardiomyocytes [72,77]. The relevance of skeletal CASQ phosphorylation in vivo, along with the effect of cardiac CASQ2 phosphorylation on the regulation of Ca^2+^ fluxes at the dyad, are still obscure aspects of CASQ biology.

### 4.3. Glycosylation

As for phosphorylation, cardiac CASQ2 in vivo exhibits a wide range of glycosylation levels, whereas skeletal CASQ1 primarily possesses a lone mannose group [72]. A paradigm for the role of glycosylation is given by research conducted on CASQ1. Significant co-translational glycosylation at a specific C-terminal Asn residue, with up to 8–9 mannose groups, hinders CASQ multimerization until the protein reaches the jSR lumen, where mannose trimming restores CASQ’s polymerization capacity so that polymerization-competent species are retained [78,79]. While bulky glycosyl chains hinder oligomerization, short mannose chains stabilize both dimerization and polymerization. This double effect is a consequence of the critical positioning of the glycosylation residue in between the C-terminus of the host monomer and the N-terminus of the dimerization partner. Mannosyl chains at the conserved Asn-316 residue hold the extended N-terminal swapped domains from one side, and physically constrain the sides of the growing polymer on the other side (Figure 4B,C) [72]. In the heart, the defective glycosylation of CASQ2 has been linked to acquired cardiac diseases; Man8,9-containing CASQ2 is highly increased in the heart failure of different etiologies, and it is worth mentioning that the protein is concomitantly more phosphorylated [80,81]. As far as genetic diseases are concerned, the mutated CASQ2-K206N (linked to Catecholaminergic Polymorphic Ventricular Tachycardia type 2) features a de novo N-glycosylation site, together with defective SR targeting and reduced Ca^2+^-binding capacity [82].

## 5. Molecular Patho-Physiological Implications of CASQ’s Defects

### 5.1. Uncertainties Surrounding the Biochemical Behavior of CASQ’s Pathological Variants

Skeletal CASQ1 pathological missense mutations in heterozygous conditions are linked to either malignant hyperthermia (CASQ1 M87T) or tubular aggregate myopathy (CASQ1 D44N, G103D, D244G, I387T) [15,83,84], whereas the numerous pathological mutations of the cardiac isoform lead to Catecholaminergic Polymorphic Ventricular Tachycardia type 2 (CPVT2) mainly in homozygosis [31,36,85]. CASQ1 and CASQ2 missense mutations are scattered across all three thioredoxin domains of CASQ and the mutated proteins do not share any common defect in their Ca^2+^-dependent polymerization properties [31,36,85,86]. Moreover, most of the published biochemical analyses on either the polymerization and/or Ca^2+^-buffering capabilities of recombinant CASQ missense pathological mutants [15,28,31,33,34,35,36,85,86,87,88,89,90] (Table 1) are sometimes difficult to interpret or in contrast with each other.

A first limitation that may have led to this situation is the previously described hyper sensitivity of CASQ to the ionic conditions [85,86]. Secondly, the very intimate relationship between Ca^2+^-binding events and polymerization complicates the interpretation of Ca^2+^-binding curves, particularly those obtained with methods such as MicroScale Thermophoresis, which are sensitive to changes in molecular size. A third limiting aspect involves the widely used turbidity assay, which cannot discriminate among multimerization kinetics and/or aggregation processes occurring in vivo. Different polymerization defects, for example, characterize CASQ1, D244G, and I385T mutants, either in their linear polymerization or in the formation of branching points [15]. In addition, the post-translational modifications of CASQs are not well accounted for in in vitro assays. On the road towards a pathophysiological understanding of CASQ mutations, it appears that the results obtained from the wealth of in vitro approaches should not be considered in isolation, but as part of a complex, still fragmented scenario.

### 5.2. Dimerization of CASQ Missense Variants May Underlie Distinct Penetrance Mechanisms

The variable penetrance of inherited CASQ2 mutations [31,36], when combined with the 100% penetrance of the dominant K180R CASQ2 [91], has recently led to the hypothesis that two distinct molecular processes, triggered by two distinct groups of CASQ2 mutations, may underlie the recessive and dominant forms of CPVT2 [31,89].

Starting from the above, we propose here the first functional classification of the pathological missense mutations of CASQs, which stems from the expected structural consequence on the stability of either the monomer, the dimer, or the recently crystallized linear polymer conformation [31] (Figure 4D, Table 1). For each characterized pathological mutation, we compiled its biochemical feature as described in published studies.

In Table 1, each column summarizes the biochemical data published for each of the most studied pathological mutants for either skeletal or cardiac CASQ. Mutants have been clustered and colored depending on the CASQ conformational state possibly affected (either the stability of the monomer, of the dimer, or of the polymer), and, within each structural group, ordered by the residue number. Lighter to darker colors reflect the quaternary state, from the monomer to the polymeric one, possibly affected by the mutation. CASQ2 pathological variants are colored from light blue to dark blue, and CASQ1 from light yellow to dark yellow, from the left to the right. Mutations altering the folding of the monomer encloses those mutation residues falling within the hydrophobic core of the thioredoxin domains, whereas mutations altering the inter-monomer dimerization or tetramerization interfaces have been included in the “Dimer”, “D/P”, or “Polymer” groups, respectively. An additional group named “P*” contains a mutants with supposed altered polymeric conformation. In the crystal structure by Titus et al. [31] (PDB ID: 6OWW) (Figure 4D), 28 was used as a model for the structural inspection and sorting of the mutations. Experiments are listed on the first column on the left and are also grouped based on the type of information given. Those experiments informing on the tertiary architecture folding and stability of the protein monomer in the absence of Ca^2+^ (tryptophan fluorescence, Circular Dichroism, degree of protection by trypsin digestion, melting temperature curve, mono-dispersion in Gel Filtration Chromatography) have been grouped under the name “tertiary structure”. Experiments analyzing the Ca^2+^-induced structural rearrangements and Ca^2+^-binding affinities are grouped under the name “Ca^2+^-dependent properties” (Ca^2+^-induced protection by trypsin digestion, Ca^2+^-induced changes in the Circular Dichroism spectrum and tryptophan fluorescence signal, Ca^2+^-induced increase in the apparent radius of gyration on Dynamic Light Scattering, appearance of a peak species in Gel Filtration Chromatography corresponding to the elution volume for a CASQ dimer, Ca^2+^-induced increase in the 350 nm turbidity, Ca^2+^-induced increase in the 350 nm turbidity of the protein pre-incubated with 2 mM Mg^2+^, Ca^2+^-induced increase in the high-molecular-weight species in Native-PAGE, Ca^2+^-binding curve measured either through MicroScale Thermophoresis or equilibrium dialysis). For each box, the experimental interpretation is summarized. More specifically, the behavior of each mutant is evaluated for each indicated experiment in terms of the overall information gained. For those experiments assessing CASQ “tertiary structure” in absence of Ca^2+^, the interpretation refers to the stability or retainment of the wild-type monomeric conformation, whereas for those experiments assessing the Ca^2+^ responsiveness, either conformational or in terms of binding kinetics, the interpretation is given in terms of the amplitude of the structural rearrangement or binding affinity relative to that of the wild-type protein in the same condition. ↓: lower than wt, =: identical to wt, ↑: higher than wt. References are given for each experiment. Additional details are given in the ‘Notes’ section.

A trend emerges revealing two groups with distinct biochemical behaviors, where the discriminant structural feature is the in vitro ability to dimerize or not (Table 1). Given that proper CASQ sorting and retention within the jSR is determined by the protein’s ability to multimerize, it has been hypothesized that those CASQ mutants unable to dimerize would not be targeted or be allowed to reside within the jSR, leading to haploinsufficiency when in heterozygosis, and to loss of CASQ when in homozygosis [31]. In accordance with this hypothesis, the only recessive CASQ2 mutations that have been characterized in murine models, sharing a consistent loss of CASQ2 within the jSR [92,93], fall (R33Q [94], D307H [95]) or would fall (G112 + 5X [96]) within the first non-dimerizing group of proteins (on the left side of Table 1). The second group is that of dimerization-competent mutants (on the right side of Table 1), which includes many of the dominantly inherited CASQ1 variants, the only CASQ2 mutation whose dominance has been clinically and functionally validated to date [89,91], and other mutations clinically associated with CPVT2 in heterozygosis [36]. In those cases, we hypothesize that the extremely subtle defects, undetectable at the dimer level, might be amplified and, thus, exert their pathological effect only within the polymer structure(s), which could present altered Ca^2+^-binding/release properties and interactome. In this regard, it is indeed interesting to note that the only mouse model carrying a mutation from the second group (CASQ2-K180R) did not show any loss in the total intra-SR calcium buffering capacity (contrary to all CPVT2 recessive models), but did show a defect in the dynamic intra-SR calcium buffering [89]. Our hypothesis points to the CASQ dimerization of pathological mutants as a critical aspect in determining the intra-SR dominant/recessive pathological effect. The altered trafficking or turnover of a non-dimerizing mutant would lead to a loss of intra-SR CASQ, which in turn affects the total Ca^2+^-binding capacity of the system. On the other hand, dimerization-competent mutants, residing in the jSR, can recruit the wild-type CASQ counterpart into less responsive CASQ-Ca^2+^ systems. Of note are two CASQ1 mutations, one correlated with malignant hyperthermia (CASQ1 M87T), and a second one involved in tubular aggregate myopathy, which have been grouped in between the non-dimerizing and the dimerizing variants because their position possibly disrupts either the dimerization or the polymerization interfaces (Table 1). We expect that our functional classification provides a starting working hypothesis for the molecular understanding of the varying penetrance of pathological CASQ variants in human diseases. From all the above, it is concluded that additional clinical in vivo and in vitro studies are necessary to assess and describe the possibly distinct pathological mechanisms between the first and the second group of mutations.

## 6. Polymeric CASQ Shares Similarities with Polyelectrolyte Systems

The complex physico-chemical nature of CASQ is the consequence of the multiplexed interactions between the high number of anionic groups exposed by CASQ’s monomers, dimers, and polymers, and free ions from the solution. A new possible approach to unveil CASQ complexity might consider its similarities with anionic self-assembling polymers [97]. These polyelectrolyte materials are ubiquitously employed by nature to organize complex processes such as numerous intra-cellular and extra-cellular highly charged systems, among them nuclear chromatin, and dynamically segregate delicate processes in membrane-less compartments [98,99]. To shed light on some underrated aspects of CASQ biology, some of the most interesting similarities with polyelectrolyte systems across comparable spatial scales and their relative biological meanings are discussed below.

### 6.1. Multiple Ionizable Groups on a Highly Flexible Substrate

Polyelectrolytes are defined by the presence of multiple charged groups on a conformationally versatile substrate. This same condition also characterizes CASQs, and it is particularly brought to the extreme at the intrinsically disordered, highly charged C-terminal tail [37]. The pervading role of this segment in determining the polymerization kinetics and the interaction with members of the CRU may lie in its ability to provide multiple weak and transient interactions in the solution at various Ca^2+^ concentrations [38,51].

### 6.2. Solvent Charges Modulate the Polymeric Architecture

Freely mobile counter-ions from the solution mask the negative charges of the polymeric phase and bridge the formation of intra- and inter-chain interactions, whose number is driven by the mobile counter-ions concentration, valence, and size [97]. The typical behavior of polyelectrolytes perfectly fits current knowledge of CASQ; its physiological polymerization is mainly triggered by Ca^2+^, whereas other ions promote either similar or opposite behaviors. Monovalent potassium and sodium ions support the folding of the same tertiary structure, yet they inhibit Ca^2+^-dependent quaternary assembly (Figure 2 and Figure 3) [4,29,51,100]. Divalent charges from transition metals (Mn^2+^, C0^2+^, Ni^2+^, Cu^2+^, Zn^2+^) promote CASQ multimerization, probably because of their typical versatile coordination mechanisms. Divalent Mg^2+^ cations, abundantly present in the jSR (1 mM free) [28] have intriguing effects: the pre-incubation of CASQ with 2 mM Mg^2+^ before the addition of Ca^2+^ either facilitates the nucleation of the soluble bodies or changes their shape towards a higher radius of gyration [26,28,31].

Multiple authors have pointed out the fact that Ca^2+^ fluxes in and out from the jSR are coupled with cyclic variations of other cationic species. For example, Refs. [101,102] showed that net K^+^ countercurrent towards the SR occurs concomitantly to RyR-mediated Ca^2+^ release in ventricular myocytes, ensuring the neutrality of the electrochemical potential differences across the SR membrane. Likewise, in skeletal muscle SR vesicles, it was long reported that K^+^ (and anion) fluxes, which balance the electrical charges accumulated in the SR during Ca^2+^ uptake, mediate changes in SR volume by generating osmotic pressure [103]. Whether and how such cyclical variations in cation levels impact the conformation of CASQ remains obscure [25,30,36,72,104].

Protons (H^+^) compete for CASQ’s Ca^2+^-binding sites [25,27,30] and can enhance in vivo the effect of a high Ca^2+^ load within the jSR [30]. While we remain unaware of a demonstration of neither beat-to-beat nor single-stimulation intra-SR pH transients in living cells, we must also note that, based on observations from skeletal muscle, intra-SR pH is believed to be in equilibrium with cytosolic pH due to a large proton permeability of the SR membrane [105]. In this regard, frequency- and β-adrenergic stimulation-dependent beat-to-beat cytosolic acidifications have been measured in the cytosol of healthy ventricular myocytes (about −0.12 pH units at 0.5 Hz in rabbits [106]). Based on all the above, it is tempting to note that, if such beat-to-beat intra-SR acidifications were present (and of sufficient strength), they would decrease Ca^2+^ binding to CASQ2 [25], aiding in the RyR recovery from refractoriness (as free intra-SR Ca^2+^ would increase faster for any given SR Ca^2+^ uptake rate) while simultaneously helping in maintaining the assembly of CASQ. Interestingly, very low pH conditions drove the crystallization of the only polymeric structure published [31].

Finally, based on the behavior of known carboxylate-rich polyanionic gels, one final issue of “macroscopic” nature must be borne in mind: the sharp volume changes in the polyelectrolyte gel would be expected to occur as the concentration of bivalent cations and pH change [97,107]. This effect seems unlikely to occur in vivo for CASQ in short time scales (i.e., for simple polyanionic gels, the time scales required are in the order of thousands of seconds [107]). However, experiments comparing such behavior in jSR vesicles filled with either WT or mutated CASQ (or a combination of both) might provide new hypotheses about how specific mutations affect the long-term structural remodeling of the jSR (a common finding in CASQ-related diseases, e.g., [94]).

### 6.3. Long-Range Conductance

**Figure 5:**
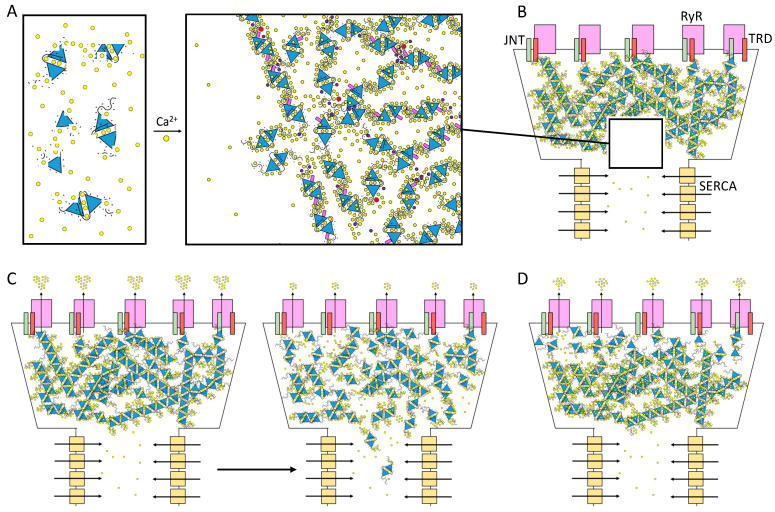
The physiological consequences of CASQ complex polymerization kinetics. (**A**) Scheme of CASQ multimerization. Multiple factors other than the concentrations of Ca^2+^ ions (yellow spheres) and of CASQ monomers (blue triangles) regulate the organization of polymeric structures. The triblock design of the CASQ dimer (two negatively charged and flexible C-termini exposed from the globular dimeric architecture) allows for the formation of a cross-linked network, where the high concentration of carboxylate groups (carried by Glu and Asp residues) sequesters the free Ca^2+^ ions from the solution. The Ca^2+^ concentration sustaining the critical polymer dimensions and shape is influenced by Mg^2+^ ions (purple) and post-translational modifications such as phosphorylation (red) and glycosylation (pink). The changes in the dimensions and shape of CASQ-Ca^2+^ nM sized bodies have usually been measured as variations in the proportion of diffracted light (the Tyndall effect for colloidal solutions). Protein partners at the jSR membrane may also play a role in shaping the orientation and architecture of the CASQ-Ca^2+^ system. (**B**) Scheme of CASQ system functioning in vivo. As revealed by multiple EM images, CASQ is a high-capacity Ca^2+^-binding polymeric matrix within the junctional SR. Ca^2+^ ions are continuously pumped in the SR from the cytosol by SERCA pumps disposed along the longitudinal SR. The opening of the RyR channels allows for the rapid efflux of Ca^2+^ ions across a very steep electrochemical gradient. The presence of CASQ sustains the outflow of Ca^2+^, giving rise to a “hump” in the Ca^2+^ release curve [16]. This pool of Ca^2+^ ions may belong to a fast-conductance reservoir that is contributed by the overall polymeric architecture and geometry. (**C**,**D**) Scheme of the possible physiological scenarios for different CASQ polymer ionic properties. (**C**) Left panel: If a rapidly disposable reservoir of Ca^2+^ ions can be conducted within the CASQ polymeric network at a speed comparable with the rate of Ca^2+^ outflow, at least the first burst of Ca^2+^ efflux might be sustained by the Ca^2+^ conductance of the CASQ system without the need for CASQ to complete any structural transition to lower Ca^2+^ capacity conformations. Right panel: As more Ca^2+^ ions are depleted from the jSR lumen, the Ca^2+^-buffering role of CASQ prevails and additional ions are released as free species in the solution, in concomitance with the changes in the polymer architecture and possible rearrangements of the protein–protein interactions at multiple sites. (**D**) If the Ca^2+^ conductance rate along the CASQ- Ca^2+^ network is insufficient to sustain calcium outflow through the Ca^2+^ channel, the behavior of those CASQ polymers just beneath the RyR channels should be the main determinant of Ca^2+^ efflux. Only in extreme calcium depletion conditions would a second layer of CASQ lying farther from the RyR channels respond.

The high mobility of soluble charges is a key feature of polyelectrolyte matrices and accounts for their typical systemic response to changes in pH and ionic conditions [107]. Likewise, Ca^2+^ ions layer all over the negative surface of CASQ, where they are flexibly coordinated by both the exposed acidic residues and free water molecules. The one-dimensional diffusion of Ca^2+^ along the CASQ surface [16,35,72,108] is coupled with proper alignment and geometry of the polymer chains [97,108]. This “Ca^2+^ wire” mechanism, proposed more than two decades ago [108], might accelerate in vivo the directional Ca^2+^ flow from the longitudinal SR (where Ca^2+^ ions are continuously pumped in from the cytosol) up to the jSR release channel (Figure 5B). Two released crystal structures of a CASQ filament beautifully fit this hypothesis; in both cases, a solvent-accessible tunnel traverses the length of the linear polymer [31,109] and is lined by several Ca^2+^-binding carboxylates. However, the very low pH conditions of the first one, published in 2020, may pose caution on its relevance as a Ca^2+^-bound state [31], whereas the second one (deposited with PDB ID: 7F05) must await formal peer-reviewed publication containing experimental details on the crystallization condition and concomitant analysis [109]. Studies on the kinetics of Ca^2+^ dissociation from CASQ revealed that its polymeric conformation is retained during the first rapid burst of Ca^2+^ release, showing that a reservoir of fast-conductance, readily available Ca^2+^ ions can be provided without any rearrangement of the polymer [104]. This in vitro observation easily correlates with the fact that the first “hump” of the Ca^2+^ release curve in vivo is determined by CASQ [16]. On these bases, it is expected that the first Ca^2+^ ions exiting the SR are not provided by RyR-neighboring CASQ proteins as single entities, but implies the participation of the polymeric CASQ as a whole (Figure 5B–D). Further studies on the Ca^2+^ conductance properties of the CASQ system, including how conductance changes as release and uptake proceed, are needed to reveal important aspects of the kinetics of Ca^2+^ outflux from the RyR channel [109]. Further insights on the nature of the possibly multiple distinct reservoirs of Ca^2+^ hosted within polymerized CASQ (Figure 5) might provide interesting clues on the CASQ-driven termination of Ca^2+^ release [41,110] through RyR.

### 6.4. Ca^2+^-Driven Membrane-Less Compartmentalization

In the sarcoplasmic reticulum lumen, CASQ is retained within the junctional space [68,111,112,113]. The diffusion of CASQ along the longitudinal SR occurs only in response to extreme Ca^2+^ depletion [16]. A first explanation for this membrane-less compartmentalization lies in the fact that 30% of CASQ [16,35,72] has been found to be physically tethered to the jSR membrane through Ca^2+^-dependent, non-covalent bonds with the Ca^2+^ Release Unit [114] (Figure 1). On the other hand, it has to be taken into account that the Ca^2+^-dependent conformational states of CASQ feature distinct solubilities, hence they may also have a role in limiting CASQ dispersion within the SR [83]. A recent manuscript described that recombinant CASQ1 can reversibly form coacervates in vitro in response to the addition of 10 mM Ca^2+^ [21]. The reversible de-mixing of homogeneous solutions into liquid compartments with distinct concentrations of a solute is typical of highly charged organic polymer/salt mixtures [97], described under the common name of “liquid-liquid phase separation (LLPS)” [99,115]. Regarding CASQ, this remains an interesting hypothesis to be further tested, especially because CASQ features a seemingly Intrinsically Disordered Region (IDR) at its C-terminus (Figure 4A), a characteristic of all known proteins involved in LLPS. The multivalent, transient network of Ca^2+^-dependent interactions that such a domain provides may indeed seed the formation of highly CASQ populated regions of the solution, where multiple CASQ proteins would hold each other in a flexible mode within a restricted space (Figure 5A). CASQ Ca^2+^-dependent jSR retainment may be a sign of its physiological behavior as an organized membrane-less system. This is a hypothesis with interesting effects on SR Ca^2+^ release that remains to be tested.

## 7. Final Remarks

Since its initial discovery by MacLennan and Wong in 1971 [5], CASQ has mesmerized those involved in its study, from muscle physiologists to biochemists and medical doctors. The first three decades following its discovery were focused on biochemistry and overall characterization. Since the early 2000s, however, with the advent of genome sequencing, transgenic animals, and the discovery of CASQ-related hereditary diseases, our biochemical understanding has progressed far more slowly, as attention has focused on the link between physiopathology and associated genetic mutations. That being said, it is our belief that understanding CASQ’s pathologically relevant defects might require a renewed discovery of its structural transitions, from monomers to cross-linked polyelectrolyte-like polymers, and of the relative kinetics for the binding of Ca^2+^ and protein partners. Within this scenario, a hypothesis has emerged according to which the dimer-to-tetramer/polymer transition is a critical aspect of CASQ physiology and pathology. New comprehensive approaches investigating the polymeric, ionic, and dynamic nature of CASQ as a system might provide unprecedented opportunities to increase our knowledge and thus our therapeutical possibilities in the fight against striated muscular disease.

## Figures and Tables

**Table 1:** Skeletal and Cardiac CASQ pathological missense mutations.

	Altered Structure:		Monomer			Dimer				D/P		Polymer					P*	
	Mutation		CASQ2 L167H	CASQ2 P329S	CASQ2 G332R	CASQ1 D44N	CASQ2 R33Q	CASQ2 D307H	CASQ2 P308L	CASQ1 M87T	CASQ1 G103D	CASQ2 S173I	CASQ2 K180R	CASQ1 D244G	CASQ2 D325E	CASQ2 D351G	CASQ2 R251H	CASQ1 I385T
Tertiary structure	Monomer stability	Tryptophan Fluorescence	↓ [33]				↓ [33]	↓ [33]										
		Circular Dichroism	↓ [28,33,90]				= [28,33,34,90]	= [28,33]	= [28]									
		Trypsin digestion protection	= [35,85]	↓ [85]	↓ [85]		↓ [85,90]	↓ [85] = [87]					= [85]			= [85]		
		Thermal stability	↓ [85]	↓ [85]	↓ [85]		= [34,85]	↓ [85]					= [85]			= [85]		
		Mono-dispersion in SEC	= [90]				↓ [36]		= [36]	= [35]		= [36]	= [36]	= [35]	= [36]		= [36]	
Ca^2+^-dependent properties	conformational rearrangement	Trypsin digestion protection	↓ [90] = [28]	↓ [28,85]	↓ [28]	↓ [15]	↓ [28,85]	↓ [28]			↓ [15]		= [85]	= [15]		= [85]		= [15]
		Secondary structureCD, TF	↓ [85,90]	↓ [28]	↓ [28]		↓ [34,85] = [90]	↓ [28]					= [85]			= [85]		
		Mass increase DLS				↓ [15]					↓ [15]			↑ [15]				↑ [15]
	dimerization	SEC-MALS		↓ [33]			↓ [33]	↓ [33]		= [35]				= [35]				
	oligomerization	Turbidity		↓ [28]		↓ [15]	↓ [28,34]	↓$ [28]	↓$ [28]	↓ [35]	↓ [15,88]	↓ [31]	= [31]	↑ [15,35]				↑ [15,88]
		Turbidity with Mg^2+^		↓ [36]			↓ [31,36]		↓ [36]			↓ [31,36]	↓ [31]		↓ [36]		=_£_ [36]	
		Native PAGE	↑§ [85]	↑ [85]	↑ [85]		↓ [85]	= [85]					= [85]			= [85]		
	Ca^2+^ binding	Microscale Thermophoresis	↓ [85]	= [85]	↓ [85]		= [85]	↓ [85]					= [85]			= [85]		
		Equilibrium dialysis	↓ [33]				↓ [33]	↓ [33]		↓ [35]				↓ [35]				

D/P: Dimer and polymer: both states could be affected. P*: Probable effect on the polymer behavior: CASQ2 R251 shapes the tunnel running within the linear polymer structure 6OWW. It may alter the kinetics of Ca^2+^-binding. CASQ1 I385 lies in close proximity to the glycosyl group in structure 3TRQ. It may alter the effect of glycosylation (Figure 4B). $ The defect appears only between 1.5 and 6 mM Ca^2+^. § Oligomers are constitutive and Ca^2+^-independent. £ Partial defect appears at pH 5.6.

## Data Availability

All data used to compile this review work have been previously published.
